# Prognostic Significance of Pan-Immune-Inflammation Value in Patients with HER2-Positive Metastatic Breast Cancer Treated with Trastuzumab Emtansine

**DOI:** 10.3390/ph17070824

**Published:** 2024-06-23

**Authors:** Taha Koray Sahin, Arif Akyildiz, Osman Talha Dogan, Gozde Kavgaci, Deniz Can Guven, Sercan Aksoy

**Affiliations:** 1 Department of Medical Oncology, Hacettepe University Cancer Institute, 06100 Ankara, Turkey; drakyildizarif@gmail.com (A.A.); drgozdekavgaci@gmail.com (G.K.); denizcguven@hotmail.com (D.C.G.); saksoy07@yahoo.com (S.A.); 2 Department of Internal Medicine, Hacettepe University Faculty of Medicine, 06100 Ankara, Turkey; osmantalhadogan@gmail.com; 3 Medical Oncology Clinic, Health Sciences University, Elazig City Hospital, 23200 Elazig, Turkey

**Keywords:** breast cancer, HER2-positive, T-DM1, PIV, pan-immune-inflammation value

## Abstract

Trastuzumab emtansine (T-DM1) is a mainstay therapy for HER2-positive metastatic breast cancer (mBC). However, identifying patients who will benefit most remains a challenge due to the lack of reliable biomarkers. The recently developed pan-immune-inflammation value (PIV), a novel immune-inflammation marker, could aid in this regard, considering the immunomodulatory effects of T-DM1. Therefore, we aimed to evaluate the association between the PIV and the efficacy of T-DM1 in patients with HER2-positive mBC. A total of 122 HER2-positive mBC patients treated with T-DM1 were included. Receiver operating characteristic (ROC) curve analyses were conducted to determine the optimal PIV threshold value for survival prediction. Kaplan–Meier survival curves and Cox regression analyses were used for univariable and multivariable survival analyses, respectively. The median age was 51 years, and 95.1% of the patients had ECOG PS 0-1. The optimal PIV cutoff value was identified as 338 in ROC analyses (AUC: 0.667, 95% CI: 0.569–0.765, *p* = 0.002). The multivariate analysis revealed that patients in the high-PIV group had significantly shorter OS (HR: 2.332; 95% CI: 1.408–3.861; *p* = 0.001) and PFS (HR: 2.423; 95% CI: 1.585–3.702; *p* < 0.001) than patients in the low-PIV group. Additionally, both ORR and DCR were significantly lower in the high-PIV group (36.6% vs. 61.3%, *p* = 0.011; 56.1% vs. 76.0%, *p* = 0.027). Our findings suggest that pre-treatment PIV may be a novel prognostic biomarker for HER2-positive mBC patients receiving T-DM1. A low PIV level is associated with more favorable outcomes. Future prospective studies are warranted to validate these findings and explore the potential utility of PIV in aiding treatment decisions.

## 1. Introduction

Human epidermal growth factor receptor-2 (HER2) gene amplification occurs in approximately 20% of invasive breast cancers and portends a poorer prognosis with higher rates of recurrence and shorter progression-free survival (PFS) and overall survival (OS) [1]. However, the emergence of HER2-targeted therapies, including tyrosine kinase inhibitors, monoclonal antibodies, and antibody–drug conjugates (ADCs) has significantly altered the therapeutic landscape for HER2-positive metastatic breast cancer (mBC) [2]. 

T-DM1 is an antibody–drug conjugate (ADC) that combines the HER2-targeting activity of the monoclonal antibody trastuzumab with the cytotoxic effect of the microtubule-disrupting agent emtansine [3]. In the phase III EMILIA trial, T-DM1 demonstrated a significant improvement in PFS (9.6 vs. 6.4 months; HR: 0.65; 95% CI: 0.55–0.77; *p* < 0.001 and OS 30.9 vs. 25.1 months; HR: 0.68; 95% CI: 0.55–0.85; *p* < 0.001) compared with capecitabine plus lapatinib for patients with HER2-positive mBC who were previously treated with trastuzumab and a taxane-based treatment [4]. Another phase III trial, TH3RESA, confirmed improvements in both PFS and OS with T-DM1 in HER2-positive mBC patients who had progressed on at least two prior HER2-targeted therapies [5].

While T-DM1 improved outcomes in the second or later lines of treatment of HER2+ mBC, the progression is inevitable for most patients, and reliable biomarkers for long-term efficacy and early progression are absent. A study by Müller et al. suggests that the benefit of T-DM1 in HER2-positive breast cancer might partly be due to immune system activation, as shown by an increase in tumor-infiltrating lymphocytes (TILs) following neoadjuvant treatment [6]. A recent study illustrates that immunogenic cell death is a key mechanism of action of T-DM1 [7]. These findings suggest that the benefit of T-DM1 might be partially facilitated through an immune response against breast cancer, and the efficacy of T-DM1 could be predicted by the reflectors of the immune-inflammatory system. The pan-immune-inflammation value (PIV), a recently established marker of tumor immunity in early and advanced breast cancer, could be a useful biomarker in this regard [8,9]. The rationale for establishing the PIV as a prognostic marker lies in the understanding of the pivotal role of inflammation in cancer progression [9]. Elevated PIV values have been associated with poorer outcomes across various cancer types, as they often indicate an enhanced inflammatory milieu [10,11,12]. However, the association between the PIV levels and survival with T-DM1 was not investigated previously. Given the absence of established prognostic or predictive biomarkers for T-DM1 therapy in HER2-positive mBC, we aimed to assess PIV’s utility as a potential indicator of treatment efficacy. 

## 2. Results

### 2.1. Baseline Characteristics

A total of 122 patients were included. The median age was 51 years (IQR 43–61) at T-DM1 initiation. Most patients (76.2%) exhibited HER2 IHC 3+, and 54.9% (n = 67) were hormone-receptor-positive (ER+ or PR+). The majority of patients had only one organ with metastasis at T-DM1 treatment initiation (65.6%); the most common were bone, brain, and lung (33.6%, 27.0%, and 24.6%, respectively). Prior to T-DM1, the percentages of patients receiving pertuzumab, lapatinib, and anthracycline therapy were 36.9%, 21.3%, and 55.7%, respectively. T-DM1 administration varied across treatment lines as a second-line (43.5%), third-line (29.5%), or later-line (27.0%) treatment (Table 1).

**Figure 1:**
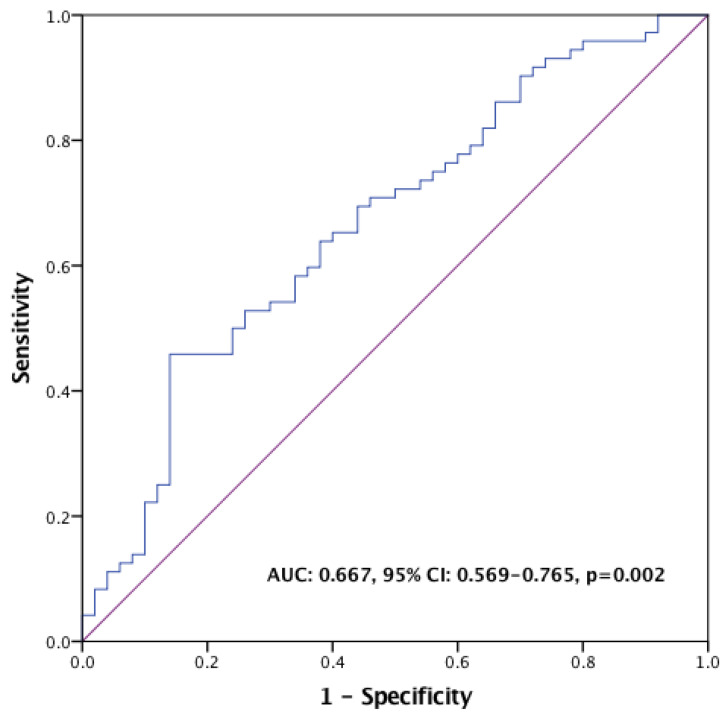
ROC curve for PIV in the prediction of overall survival.

The ROC analysis with OS as the endpoint indicated that the optimal PIV cutoff value was 338 (AUC: 0.667, 95% CI: 0.569–0.765, *p* = 0.002) (Figure 1). Patients were categorized into the low-PIV and high-PIV groups based on this PIV value. Demographics and baseline characteristics were balanced for both groups, except for a significantly higher proportion of patients in the high-PIV group receiving pertuzumab prior to T-DM1 treatment compared to the low-PIV group (*p* = 0.044) (Table 2).

### 2.2. Survival Outcomes

**Figure 2:**
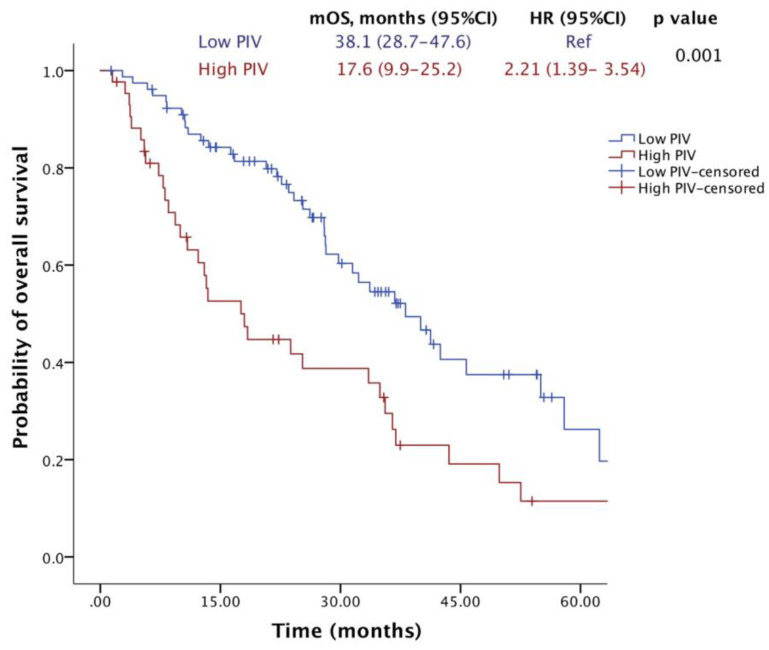
Overall survival of patients receiving T-DM1 according to PIV levels.

**Figure 3:**
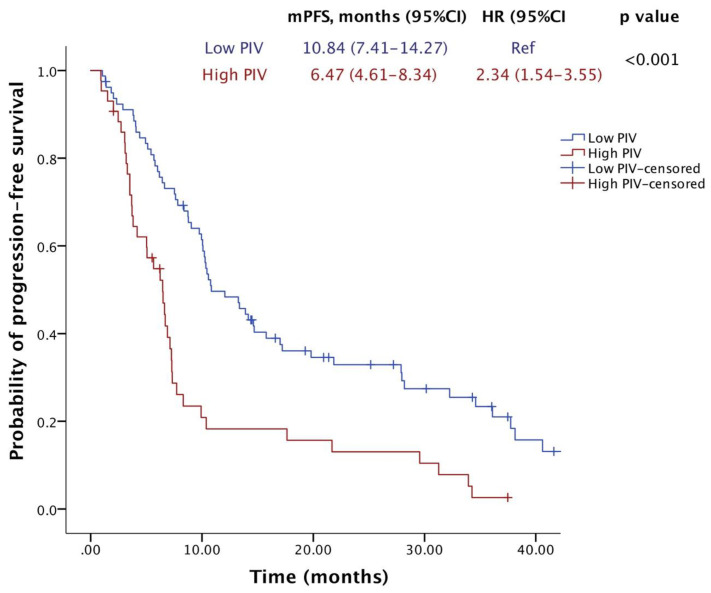
Progression-free survival of patients receiving T-DM1 according to PIV levels.

After a median of 23.4 months of follow-up, 72 (59%) patients died, and 100 (82%) patients had a PFS event. The median OS was 33.5 months (95% CI, 26.1–40.9), and the median PFS was 9.7 months (95% CI, 7.7–11.8). Notably, the median OS was significantly lower in the high-PIV group (17.6 months) compared to the low-PIV group (38.1 months) (HR = 2.21; 95% CI: 1.39–3.54; *p* = 0.001) (Figure 2). Similarly, patients with high PIV levels who were treated with T-DM1 exhibited a shorter median PFS (6.47 months) compared to those with low PIV levels (10.84 months) (HR = 2.34; 95% CI: 1.54–3.55; *p* < 0.001) (Figure 3).

In addition to higher PIV levels, higher NLR levels (high vs. low, *p* = 0.023), higher ECOG score (0 vs. 1–2, *p* = 0.005), receiving T-DM1 as a later line of treatment (≥2 vs. <2, *p* = 0.017), and prior use of pertuzumab (*p* = 0.047) were significantly associated with a worse OS in univariate analyses (Table 3). Similarly, a higher number of metastatic sites (≥2 vs. <2, *p* = 0.001), receiving T-DM1 as a later line of treatment (≥2 vs. <2, *p* = 0.010), and higher PIV levels (*p* < 0.001) were significant factors for PFS in univariate analyses. In multivariate analyses, patients with higher PIV levels (HR: 2.332, 95% CI: 1.408–3.861, *p* = 0.001), a higher number of metastatic sites (HR: 1.603, 95% CI 1.026–2.685, *p* = 0.046), and higher ECOG status scores (HR 2.726, 95% CI: 1.628–4.564, *p* < 0.001) had decreased OS. As for PFS, higher PIV levels (HR 2.423, 95% CI 1.585–3.702, *p* < 0.001) and receiving T-DM1 in a later line (HR 1.735, 95% CI 1.141–2.637, *p* = 0.010) were significant independent factors in multivariable analysis (Table 4).

### 2.3. Radiological Responses 

**Figure 4:**
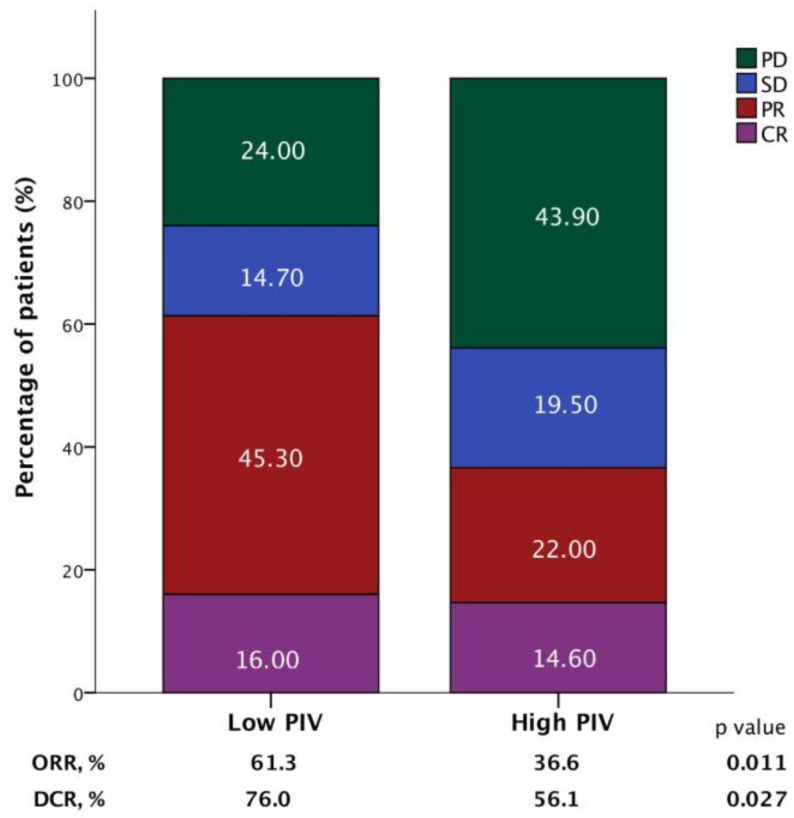
Bar graphs demonstrating the association between the objective response rate (ORR) and disease control rate (DCR) according to the pan-immune-inflammation value (PIV) in patients receiving T-DM1.

The ORR was 52.6% in the overall population; 18 patients (15.5%) experienced CR, and 43 patients (36%) experienced PR. Another 16.4% of patients achieved SD, which corresponds to a DCR of 69%; 31% had progressive disease (PD) as their best response. The high-PIV group exhibited significantly lower ORR and DCR compared to the low-PIV group (36.6% vs. 61.3%, *p* = 0.011; 56.1% vs. 76.0%, *p* = 0.027) (Figure 4).

## 3. Discussion

In the present study, we demonstrated that lower baseline PIV levels were significantly associated with improved OS and PFS compared to higher PIV levels. This association was observed independently of age, hormone receptor status, or HER2 status. We also demonstrated a statistically significant association with the treatment response, as evidenced by higher ORR and DCR in the low-PIV group compared to the high-PIV group. To the best of our knowledge, this study represents the first study to evaluate the association between PIV level and survival outcomes in patients with HER2-positive mBC receiving T-DM1.

Following the pivotal phase III trials EMILIA [4] and TH3RESA [5], which established T-DM1 as a standard therapeutic option for HER2-positive breast cancer, this single-center retrospective analysis offers valuable real-world data on treatment effectiveness. Our study population demonstrated a median PFS of 9.7 months, which is comparable to the 9.6 months reported in the EMILIA study and longer than the 6.2 months reported in the TH3RESA study. Similarly, the observed OS of 33.5 months surpassed the 30.9 months and 22.7 months reported in EMILIA and TH3RESA, respectively. This disparity in OS might be attributable to potential differences in the prior lines of therapy received by patients enrolled in these trials. Although T-DM1 has become a promising treatment for HER2-positive breast cancer, the factors affecting its effectiveness are still unclear. 

Trastuzumab deruxtecan (T-DXd) is a recently developed ADC incorporating a humanized anti-HER2 antibody, a peptide-based cleavable linker, and a novel potent topoisomerase I inhibitor payload [13]. The DESTINY-Breast03 trial demonstrated a significant improvement in both PFS and OS with T-DXd compared to T-DM1 in patients with HER2-positive mBC who had previously been treated with trastuzumab and a taxane [14]. These findings led to FDA approval of T-DXd as a second-line treatment of adult patients with HER2-positive mBC. Despite demonstrating superior efficacy in trials, limited national health insurance coverage restricts access to T-DXd, making T-DM1 the more frequently utilized second-line therapy in clinical practice. It is also important to consider the toxicity profiles of these treatments; T-DXd has been associated with significant adverse effects, including interstitial lung disease (ILD) and pneumonitis, which require vigilant monitoring due to their potential severity. In contrast, T-DM1 generally exhibits a more favorable toxicity profile, with most adverse events being manageable and less severe, such as thrombocytopenia and elevated liver enzymes. This difference in toxicity profiles is crucial when evaluating the overall risk–benefit ratio of these therapies for individual patients. 

The significant correlation between a high HER2 expression level and improved T-DM1 efficacy is well documented and appears likely to be mediated by increased HER2-mediated endocytosis and subsequent lysosomal degradation of the antibody–drug conjugate [15,16]. This mechanism potentially leads to higher intracellular concentrations of the cytotoxic payload, DM1, within HER2-overexpressing cancer cells [16]. High HER2 mRNA levels were found to be associated with better tumor responses and PFS in the metastatic setting treated with T-DM1 [17]. Interestingly, Baselga et al. revealed a more pronounced effect of T-DM1 on OS compared to PFS in patients with high HER2 mRNA levels [16]. This observation suggests that T-DM1 may exert its anti-tumor effects beyond direct HER2 targeting.

The therapeutic landscape for cancer is shifting towards a deeper understanding of the interplay between the anti-tumor immune response and direct cytotoxic effects of anti-cancer therapies [18]. Tumor-infiltrating lymphocytes (TILs) are a readily obtainable biomarker of the intra-tumoral immune response and are gaining interest as promising predictors of treatment response [19]. The KRISTINE phase III study demonstrated a positive association between TIL infiltration and the response to T-DM1 treatment [20]. Moreover, the KRISTINE study revealed that higher expressions of both HER2 and immune markers within the tumor microenvironment correlated with higher pCR rates, suggesting that T-DM1 sensitivity might be linked to the activation of the anti-tumor immune response [20]. Additionally, a recent study demonstrated that immunogenic cell death induction is one of the mechanisms of T-DM1 sensitivity in vitro and in vivo [7]. Collectively, this growing body of evidence underscores the possibility that T-DM1’s efficacy may be partially mediated by immunity.

Although pre-clinical data suggest that the T-DM1’s anti-tumor mechanism involves immune system activation, the clinical utility of peripheral blood immune cell subsets as prognostic markers in HER2-positive mBC patients receiving T-DM1 therapy remains to be elucidated. The evaluation of the neutrophil–lymphocyte ratio (NLR) or platelet–lymphocyte ratio (PLR) in the HER2-positive breast cancer subgroup did not identify a statistically significant correlation with the clinical outcomes [21]. Similarly, Ulas et al. reported no such significant association between NLR or PLR and survival outcomes in HER2-positive BC patients treated with adjuvant trastuzumab [22]. Conversely, Imamura et al. observed a significant correlation between elevated baseline NLR and worse PFS and OS in HER2-positive mBC patients treated with second line T-DM1 [23]. This finding is intriguing, especially considering the potential of T-DM1 to induce immune activation, as evidenced by a significant increase in lymphocytes following T-DM1 initiation in patients with low NLR. These conflicting findings underscore the need for further research to definitively establish the prognostic utility of the blood cell parameters HER2+ mBC, and these findings may ultimately contribute to unraveling the mechanism and potentially aid in patient selection for optimal treatment benefit for T-DM1.

Emerging evidence suggests the PIV as a novel promising predictor of clinical outcomes in cancer patients [24]. Fuca et al. demonstrated that PIV showed a robust association with both OS and PFS in advanced colorectal cancer, and PIV’s prognostic capacity appears to surpass that of other well-established inflammatory-immune markers, such as NLR and PLR, possibly due to the inclusion of four different peripheral blood cell indices. A recent meta-analysis encompassing 15 studies and approximately 5000 patients found a significant association between elevated PIV and worse OS (HR = 2.00, 95% CI: 1.51–2.64) and PFS (HR = 1.80, 95% CI: 1.39–2.32) in cancer patients [10]. Compared to individual blood cell parameters, PIV can provide a more comprehensive reflection of the complexity of the immune landscape and various cellular components, each potentially reflecting and regulating distinct aspects of anti-tumor immunity [25].

Peripheral blood lymphocyte levels may reflect an immune reaction or potential immunity against tumor-associated antigens [26]. In contrast, neutrophils impede anti-tumor immunity through the release of different pro-tumorigenic cytokines, growth factors, and chemokines, fostering tumor progression via mechanisms such as neo-angiogenesis, metastasis, and the creation of an immunosuppressive tumor microenvironment [27,28]. Intriguingly, macrophages potentially suppress the anti-tumor immune response of T lymphocytes by releasing chemokines, contributing to the immunosuppressive state [29]. Collectively, neutrophils, monocytes, and platelets exhibit a pro-inflammatory signature in the peripheral blood of cancer patients, potentially reflecting the host’s inflammatory response to the tumor. In contrast, lymphocyte levels may serve as a biomarker for the immune system’s modulatory potential during cancer therapy. Since PIV integrates both pro-tumor and anti-tumor factors within the TME, it has the potential to serve as a surrogate marker for the degree of immunosuppression. A growing body of evidence implicates that a high PIV may represent an immune-suppressive state in the TME. We therefore postulated that T-DM1 efficacy may be higher in patients with a low PIV, which reflects lower immune suppression, and T-DM1-associated immune induction may be expected. Consistent with this hypothesis, we found a robust and independent impact of baseline PIV on survival outcomes, with higher PIV levels predicting worse PFS and OS outcomes in patients receiving T-DM1 therapy.

The present study has certain limitations. First, the single-center and retrospective design of the study restricts the generalizability of the results to the entire HER2-positive mBC population. Additionally, the retrospective data collection approach introduces the possibility of selection bias, potentially influencing the observed correlations between PIV and clinical outcomes. Our analysis did not include TILs, which were previously found to predict the clinical outcomes in the CLEOPATRA trial [30]. Finally, the optimal cutoff value for PIV remains debated, but our study chose a more stringent methodology for cutoff selection to enhance accuracy. However, despite these limitations, a robust association between baseline PIV levels and survival outcomes was observed.

## 4. Materials and Methods

### 4.1. Patient Population

Patients diagnosed with HER2-positive mBC who received T-DM1 between January 2013 and December 2023 at Hacettepe University Oncology Hospital were enrolled in this study. HER2 positivity was defined by an immunohistochemistry (IHC) score of 3 or fluorescence in situ hybridization (FISH) positivity with an IHC score of 2, as per the ASCO CAP guidelines. Patients were followed for survival data until 21 March 2024, until either the date of the last available patient record or censored date. Patients with missing clinical data were excluded.

Baseline characteristics including demographic features, Eastern Cooperative Oncology Group (ECOG) performance status, HER2 status, hormonal status, metastatic sites at diagnosis, prior lines of therapy before T-DM1 (trastuzumab, pertuzumab, lapatinib, antracycline, and taxane), and baseline laboratory parameters were collected along with the survival data. Pre-treatment complete blood count (CBC) data used for PIV calculation were collected within the week preceding T-DM1 therapy initiation. The PIV was calculated using the established formula: [monocyte count (10^3^/mL) × neutrophil count (10^3^/mL) × platelet count (10^3^/mL)]/lymphocyte count (10^3^/mL) [31].

This study was approved by the Hacettepe University Institutional Review Board (Approval Number: SBA 24/456) and conducted in line with ethical standards established in the Helsinki Declaration and its subsequent amendments. All applicable local and national regulations were complied with in the study.

### 4.2. Statistical Analyses

Continuous variables were expressed as median and interquartile range (IQR), while categorical variables were summarized as percentages and frequencies. The optimal PIV cutoff value for predicting survival outcomes was determined by Youden’s J index from receiver operating characteristic (ROC) curve analysis [32]. Based on this cutoff value, patients were divided into high-PIV and low-PIV groups. The comparison of patient characteristics between the PIV groups involved using the Mann–Whitney U test for continuous data and Fisher’s exact test or chi-square test for categorical variables.

PFS was defined as the time from first T-DM1 administration to disease progression or death, whichever occurred first. OS was defined as time from the first T-DM1 administration to last follow-up and/or death. The assessment of tumor response followed the Response Evaluation Criteria in Solid Tumors (RECIST) guideline version 1.1 [33]. The overall response rate (ORR) was the proportion of patients achieving a complete response (CR) or partial response (PR) as their best overall response. The disease control rate (DCR) was the proportion of patients achieving CR, PR, or stable disease (SD) as the best overall response. Kaplan–Meier analyses were used to estimate survival analysis, and the log-rank test compared survival times between prognostic subgroups. Multivariable Cox proportional hazards models quantified the independent effects of prognostic factors on survival with hazard ratios (HRs) and 95% confidence intervals (CIs). The multivariable survival models were constructed with the inclusion of parameters with p values below 0.10 in the univariate analyses via backwards variable selection. Data were analyzed with SPSS, version 25.0 (IBM Corp., Armonk, NY, USA). A *p* value of less than 0.05 was considered significant.

## 5. Conclusions

In conclusion, our findings suggest that baseline PIV levels may serve as a predictive marker for T-DM1 efficacy. Further prospective studies are required to confirm these findings, elucidate the mechanism, and ultimately optimize patient selection for improved clinical outcomes.

## Figures and Tables

**Table 1:** Baseline patient characteristics of study cohort (n = 122).

Characteristics	n, (%)
Age at T-DM1 treatment, median (IQR)	51 (43–61)
Hormone receptor status, n (%)	
ER+ or PR+	67 (54.9)
ER− and PR−	55 (45.1)
HER2 status, n (%)	
IHC3+	93 (76.2)
IHC2+ and ISH+	29 (23.8)
Disease extent at diagnosis, n (%)	
Early	66 (54.1)
Metastatic	56 (45.9)
ECOG performance status, n (%)	
0	55 (45.1)
1	61 (50.0)
2	6 (4.9)
Distribution of distant metastatic sites at initiation of T-DM1, *n* (%)	
Bone	41 (33.6)
Liver	20 (16.4)
Brain	33 (27.0)
Lung	30 (24.6)
Number of sites of distant metastasis at T-DM1 initiation, *n* (%)	
1	80 (65.6)
2	32 (26.2)
3+	10 (8.2)
Prior line of therapy	
Anti-HER2 therapy	
Trastuzumab	121 (99.2)
Pertuzumab	45 (36.9)
Lapatinib	26 (21.3)
Chemotherapy	
Anthracycline-based	68 (55.7)
Taxane-based	106 (86.9)
T-DM1 treatment line in metastatic setting, n (%)	
Second	53 (43.5)
Third	36 (29.5)
Fourth or more	33 (27)

**Table 2:** Comparisons of patient characteristics in the low-PIV and high-PIV groups (n = 122).

Characteristics	Low-PIV Group (n = 79)	High-PIV Group (n = 43)	*p* Value
Age group, n (%)			0.186
<60 years	57 (72.2)	26 (60.5)	
≥60 years	22 (27.8)	17 (39.5)	
Hormone receptor status, n (%)			0.319
ER+ or PR+	46 (58.2)	21 (48.8)	
ER− and PR−	33 (41.8)	22 (51.2)	
HER2 status, n (%)			0.152
IHC3+	57 (72.2)	36 (83.7)	
IHC2+ and ISH+	22 (27.8)	7 (16.3)	
Disease extent at diagnosis, n (%)			0.779
Early	42 (53.2)	24 (55.8)	
Metastatic	37 (46.8)	19 (44.2)	
ECOG performance status, n (%)			0.851
0	35 (44.3)	20 (46.5)	
1–2	44 (55.7)	23 (53.5)	
Distribution of distant metastatic sites at initiation of T-DM1, *n* (%)			
Bone	25 (31.6)	16 (37.2)	0.534
Liver	15 (19.0)	5 (11.6)	0.443
Brain	22 (27.8)	11 (25.6)	0.788
Lung	18 (22.8)	12 (27.9)	0.530
Number of sites of distant metastasis at T-DM1 initiation, *n* (%)			0.127
<2	55 (69.6)	24 (55.8)	
2 or more	24 (30.4)	19 (44.2)	
Prior line of therapy			
Anti-HER2 therapy			
Trastuzumab	79 (100)	42 (97.7)	0.352
Pertuzumab	24 (30.4)	21 (48.8)	0.044
Lapatinib	15 (19.0)	11 (25.6)	0.488
Chemotherapy			
Anthracycline-based	45 (66.2)	23 (53.5)	0.712
Taxane-based	69 (87.3)	37 (86.0)	0.840
T-DM1 treatment line in metastatic setting, n (%)			0.903
<3	34 (43)	19 (44.2)	
3 or more	45 (57)	24 (55.8)	

**Table 3:** Univariable and multivariable cox regression analyses for OS.

	Univariable			Multivariable		
Variable	HR	95% CI	*p*	HR	95% CI	*p*
Age (≥60 vs. <60)	1.403	0.868–2.270	0.167			
ECOG status (>0 vs. 0)	1.996	1.235–3.226	0.005	2.726	1.628–4.564	<0.001
Hormone receptor, positive (yes vs. no)	1.228	0.767–1.966	0.393			
HER2 status (IHC 2+/ISH + vs. IHC 3+)	1.226	0.692–2.173	0.485			
CNS metastasis (yes vs. no)	1.377	0.832–2.278	0.214			
Liver metastasis (yes vs. no)	1.404	0.782–2.524	0.256			
Bone metastasis (yes vs. no)	1.270	0.775–2.079	0.343			
Number of metastatic sites (≥2 vs. <2)	1.545	0.957–2.493	0.075	1.603	1.026–2.685	0.046
Treatment line of T-DM1(≥2 vs. <2)	1.804	1.113–2.926	0.017	1.325	0.775–2.265	0.303
Prior pertuzumab use	1.714	1.022–2.876	0.047	1.358	0.780–2.363	0.279
NLR (high vs. low)	1.748	1.080–2.827	0.023	1.590	0.939–2.693	0.084
PLR (high vs. low)	1.554	0.966–2.499	0.069	1.239	0.745–2.059	0.408
PIV value (high vs. low)	2.216	1.388–3.539	0.001	2.332	1.408–3.861	0.001

**Table 4:** Univariable and multivariable cox regression analyses for PFS.

	Univariable			Multivariable		
Variable	HR	95% CI	*p* Value	HR	95% CI	*p* Value
Age (≥60 vs. <60)	0.978	0.641–1.491	0.916			
ECOG status (>0 vs. 0)	1.290	0.867–1.919	0.209			
Hormone receptor, positive (yes vs. no)	1.352	0.908–2.014	0.138			
HER2 status (IHC 2+/ISH + vs. IHC 3+)	0.905	0.570–1.436	0.671			
CNS metastasis (yes vs. no)	0.992	0.646–1.524	0.972			
Liver metastasis (yes vs. no)	1.381	0.835–2.283	0.209			
Bone metastasis (yes vs. no)	1.450	0.948–2.217	0.086	1.120	0.673–1.866	0.662
Number of metastatic sites (≥2 vs. <2)	1.831	1.212–2.767	0.004	1.435	0.952–2.163	0.085
Treatment line of T-DM1(≥2 vs. <2)	1.716	1.139–2.585	0.010	1.735	1.141–2.637	0.010
Prior pertuzumab use	1.475	0.971–2.240	0.068	1.159	0.748–1.196	0.509
NLR (high vs. low)	1.300	0.873–1.935	0.197			
PLR (high vs. low)	1.207	0.811–1.797	0.353			
PIV value (high vs. low)	2.341	1.545–3.548	<0.001	2.423	1.585–3.702	<0.001

## Data Availability

The data that support the findings of this study are available from the corresponding author upon reasonable request.
